# Editorial: Recent Advances in Thyroid Surgery

**DOI:** 10.3390/jcm11237233

**Published:** 2022-12-06

**Authors:** Gregorio Scerrino, Pierina Richiusa, Giuseppa Graceffa, Eleonora Lori, Salvatore Sorrenti, Nunzia Cinzia Paladino

**Affiliations:** 1 Unit of Endocrine Surgery, Department of Surgical Oncological and Oral Sciences, Policlinico “P. Giaccone”, University of Palermo, Via Liborio Giuffré 5, 90127 Palermo, Italy; 2 Department of Health Promotion Sciences Maternal and Infantile Care, Internal Medicine and Medical Specialties (PROMISE), Section of Endocrinology, University of Palermo, Via Del Vespro 129, 90127 Palermo, Italy; 3 Unit of General and Oncology Surgery, Department of Surgical Oncological and Oral Sciences, Policlinico “P. Giaccone”, University of Palermo, Via Liborio Giuffré 5, 90127 Palermo, Italy; 4 Department of Surgery, “Sapienza” University of Rome, Viale Del Policlinico 155, 00161 Rome, Italy; 5 Department of General Endocrine and Metabolic Surgery, Conception Hospital, Aix-Marseille University, 147, Boulevard Baille, 13005 Marseille, France

Thyroid surgery has been, since its earliest application, one of the most notable fields in medicine, illustrated by the fact that the Nobel Prize in Medicine was won, for the first time, for thyroid surgery by Emil Theodor Kocher (1841–1917) in 1909, for his contributions to thyroid physiology, pathology, and surgery [1].

Thyroid surgery developed over the course of three centuries: from the 19th century, when it originated, thyroid surgery went through a stabilization phase and a huge innovation phase, due to continuous technological improvements. During the first two historical stages, the main advances were due to the improvement of anesthesia protocols, infection prophylaxis and basic hemostatic procedures. Another huge advance in surgery was the standardization of the surgical technique based on the main step described by Kocher.

Recently, there have been numerous improvements in this field. The first in both time and importance is safety, as the better the safety the lower the chance of complications [2]. If we take into consideration the three main complications of thyroid surgery (bleeding, hypocalcemia, and laryngeal nerve palsy), we can see that the products introduced by pharmaceutical and technological companies have helped a lot in controlling their incidence.

If continuous bleeding occurs in a narrow space, it could cause a compressive hematoma and a venous obstruction. At the same time, even reduced amounts of blood may occult small anatomical structures, such as parathyroids or laryngeal nerves. Moreover, compartmentalized collections of blood can eventually evolve into a superinfection. Considering the aforementioned complications, it is evident that the optimization of hemostasis is a primary goal in thyroid surgery. Assuming the surgical technique is accurate, there are two possible useful tools: advanced topical hemostatic agents and energy-based hemostatic devices. A considerable number of recent studies have investigated the efficacy of topical hemostatic agents in general and specifically in thyroid surgery. There are several types of formulations for hemostatic agents that can be roughly split into two categories: fluid (gel) and solid (gauze and patches). It seems that current studies agree on the following result: advanced hemostatic patches, which determine covalent bonds with wet tissues, and active patches, formulated with added biochemical agents favoring coagulation (fibrinogen, thrombin, etc.) seem preferable to hemostatic gauzes (“passive” hemostatic patches), which are capable of compression only, due to the low number of side effects and improved efficacy [3,4,5]. Due to the rarity of the compressive hematoma, recent studies have not been able to demonstrate the efficacy of the currently available agents. Nonetheless, said studies seem to agree that the agents reduce the drain output, length of stay, and, in some cases, the duration of surgery [6,7]. The use of energy-based surgical tools, such as radiofrequency, ultrasound or the combination of both, also appears to have improved many outcomes of thyroidectomy. Complications seem unchanged or even reduced but using these tools saves time. Moreover, there is no clear proof of a reduction in the incidence of major hemorrhages [8].

Hypocalcemia, the most frequent sequelae of thyroidectomy, can prolong hospitalization and postdischarge follow-up with economic and health consequences. Moreover, some evident forms can be considered persistent complications, potentially with severe long-term consequences. Once again, the correct assessment of the vascular supply of the parathyroid glands and meticulous surgical technique are crucial for optimizing the results [9,10]. In order to successfully treat hypocalcemia, calcium and vitamin D treatment needs to be properly managed. Another useful tool is the fluorescence technique, recently introduced for assessing parathyroid perfusion during thyroidectomy [11]. Moreover, distinctive autofluorescence has also been observed in parathyroid glands. This feature has allowed the surgical technique to be simplified. Currently, it is believed that autofluorescence can be used to improve the identification of parathyroid glands in situ, reducing the number of inadvertent parathyroidectomies and improving outcomes in terms of transient hypocalcemia [12]. Albeit there are two different application principles, the methods of using fluorescence in thyroid surgery seem to be promising for reducing post-thyroidectomy hypocalcemia.

The routine identification of laryngeal nerves is the gold standard to reduce post-thyroidectomy voice complications. The lateral to medial traction of the thyroid lobe is a necessary step to expose the inferior laryngeal nerve (RLN), but during this procedure the nerve can be injured without any structural changes being observable to the naked eye. This lesion depends on the stress caused by the dense fibrous tissue that keeps the thyroid gland attached to the trachea, and is situated laterally to the nerve fibers. In other cases, thermal and/or electrical shock, clamping or other mechanical trauma, ischemia and, rarely, transection may be the cause of nerve palsy and vocal cord disfunction. The likelihood of full functional recovery depends on the causative event. If the paralysis affects only one vocal cord, the result is dysphonia of varying severity. In the case of bilateral palsy, if in adduction, there may be a reduction in the respiratory space so severe that prolonged intubation or even tracheostomy could be required, which may be a traumatic experience for patients. Originally, the goal of the electromyographic monitoring of the laryngeal nerves (IONM) was to aid the surgeon in the identification of the RLN. It is now very helpful in preventing bilateral palsy thanks to the principle of “two-staged thyroidectomy”. Today, IONM is a practice that has become ordinary in high-volume endocrine or thyroid surgery centers. Originally, this technique was proposed with an “on demand” stimulation system by the operator. Then, stimulation protocols were standardized for the vagus nerve and RLN, with the purpose of establishing the adequacy of the electromyographic waveform in terms of amplitude and latency. This technique is called intermittent monitoring (i-IONM). Recently, a technique based on the continuous monitoring (c-IONM) of the vagus nerve was introduced, making it possible to rectify the potentially damaging maneuvers before they cause nerve injuries. The c-IONM technique is constantly evolving, and we have seen particular improvements in the criteria for interpretation and standardization [13].

Along with safety, the current needs of thyroid surgery are related to the growing demand to reduce invasiveness. This concept related to purely cosmetic needs; conversely, it tends to unify the lower visual impact of surgery with the reduction in symptoms related to it, and to the sequelae and complications that may accompany it. Invasiveness is not only related to a cosmetic need, such as the lower visual impact of surgery, but it also takes into consideration the reduction in sequelae and complications that may accompany such surgeries. Towards the end of the last century, the the minimally invasive video-assisted thyroidectomy (MIVAT) technique was developed in Italy, and it spread worldwide within a few years [14]. This technique follows Kocher’s original steps, minimizing dissection [15]. MIVAT has demonstrated, over the years, a significant improvement in cosmetic results and a reduction in postoperative pain, with an unchanged incidence of complications [16]. The distinctive anatomical location of the thyroid glands did not facilitate the development of additional minimally invasive techniques, which would fully meet the essential requirements of this approach.

Especially in this field, there is a dichotomy between finding the least invasive surgical techniques, by removing scars in the neck but at the cost of remote access, and a conservative technique that preserves the gland by only ablating one or more nodules, either by energy or with chemical effect.

Remote access thyroidectomy techniques were designed with the intent to abolish the anterior cervical scar, which is a cosmetic limitation even after MIVAT. The remoteness of the access from the surgical site refutes its minimally invasiveness, yet these techniques have found appeal in the possibility of leaving a completely intact neck image. Out of the different techniques proposed, trans-axillary and trans-oral methods have found wider application, while mammary or retro-auricular techniques are still in limited use. “Hybrid” techniques, such as axillo-breast and submentorial techniques, are even more rarely applied. The use of robotic solutions has improved many of these techniques, especially in terms of the accuracy of dissection and image magnification. For example, robot usage has allowed single axillary access to be possible in total thyroidectomy.

Overall, remote access compared to the standard presents a variable increase in the duration of the procedure and an increase in costs. The incidence of complications is still being evaluated, while the occurrence of new access-related complications must be taken into account. Conceivably, in the coming years more robust clinical data will lead to a definitive validation of these techniques and, possibly, an extension of applications [17].

Based on current knowledge, energy-based ablations are useful options for solid nodules. The different ablation techniques (laser, radiofrequency and, more recently, microwave) showed efficacy and safety in the treatment of toxic and non-toxic nodules. Percutaneous ethanol injections are recommended for recurrent cystic lesions. The literature encourages pursuing this direction. In fact, several clinical trials and meta-analyses have proved the efficacy and safety of ablation techniques. Due to this corroboration, they have become part of the options suggested by several guidelines in different cultural settings [18]. The use of techniques that leave out surgical excision, and consequently forgo obtaining a histologic report, is all the more attractive the less the patient is fit for surgery. Moreover, surgery for benign lesions aims to reduce the mass effect; therefore, the ablative method should also be taken into consideration. Due to the dual application of ablation, this technique should always be taken into account in a multidisciplinary setting [19].

Further developments in surgical thyroidology seem to lie ahead in fields not distinctly related to technical aspects but more to those of strategy. Today, much attention is being paid to the impact that every medical act has on quality of life. As of today, a high number of patients with undetermined cytology undergo a thyroidectomy for biopsy purposes only. Taking into consideration the high number of procedures with this characteristic, finding a better solution that respects the aforementioned needs is necessary.

To preserve the quality of life of patients, molecular biology and radiology techniques have been continuously improved over the years, in order to identify patients who can benefit from surgery or more conservative techniques.

In this sense, the development of molecular biology in the coming years could help limit the unnecessary thyroidectomies as much as possible [20]. At the same time, help in limiting unnecessary surgery has come from diagnostic imaging techniques that have grown exponentially in recent years: starting from ultrasound alone, passing through contras enhanced ultrasonography and elastosonography, up to radiomics and the use of artificial intelligence [21,22,23]. In addition, voice and swallowing disorders, difficulty in keeping body weight in check, and asthenia after thyroidectomy are extremely interesting points that may open significative lines of research, in fields that have been little explored. For these reasons, the personalized approach is necessary for the surgeon in order to identify the best therapeutic approach and avoid unnecessary complications, with the aim of preserving the patient’s quality of life [24].

We conclude by saying that, both in clinical practice and in research, the aims of thyroid surgery in the future should be: improving outcomes, reducing complications and improving quality of life.
